# Pattern of eye casualty clinic cases

**DOI:** 10.1186/1755-7682-1-13

**Published:** 2008-07-26

**Authors:** Ehab I Wasfi, Randeep Sharma, Emma Powditch, Alaa A Abd-Elsayed

**Affiliations:** 1 Eye department, Assiut University Hospital, Assiut University, Assiut, Egypt; 2 Eye Unit, Great Western Hospital, Swindon, UK; 3 Public Health and Community Medicine Department, Faculty of Medicine, Assiut University, Assiut, Egypt

## Abstract

**Introduction/Background:**

The purpose of the eye casualty clinic (ECC) is to manage patients with ocular emergencies, however a large number of patients attended the eye casualty clinic did not have an acute problem and could have been treated by their General Practitioner (GP) or referred to the eye outpatient clinic.

**Aim:**

To identify the number of patients attending the ECC every day and their route of referral and to estimate the number of patients who could have be seen and managed by a competent ophthalmic nurse practitioner.

**Methods:**

A retrospective analysis was conducted using the notes and history of all patients who attended the eye casualty clinic at the Princess Margaret Hospital in Swindon during two weeks in March 2006.

**Results:**

The average daily attendance was 21 patients who were seen between morning and afternoon sessions in the Eye Casualty Clinic.112 (54%) patients were female. The median patient age was 50 years with an age range of 1 to 91 years. 68 (34.2%) patients attended as self referrals without GP letters as our eye casualty clinic is open to the general public from 9.00 a.m. to 5.00 p.m. A & E referred 28 (14.1%) patients of which only 3 had a General Practitioner (GP) letter and only 1 patient had a walk-in centre letter. There was insufficient information to assess whether 14 patients could have been managed by a nurse; of the remaining 195 visits, 50 (25.6%) patients could have been managed by an Ophthalmic Nurse Practitioner and 145 (74.4%) patients could not have been managed by an Ophthalmic Nurse Practitioner.

**Conclusion:**

The workload of the eye casualty doctors could be decreased by 38.6% if defined categories of patients were managed by the ophthalmic nurse practitioner, appropriate referrals were directed to the General Clinic and casualty patients were not followed up inappropriately.

## Introduction/Background

The purpose of the ECC is to manage patients with ocular emergencies, however a large number of patients attending the eye casualty clinic do not have an acute problem and could have been treated by their GP or referred to the ECC.

This has resulted in an increase in the number of patients attending the casualty clinic and a lack of spaces to book reviews into the casualty clinic. This leads us to question how we are receiving referrals and the clinical management of these patients. We are also prompted to explore ways to decrease the number of patients seen by the doctor in the ECC without compromising the quality of patient care.

Department of Health, 2002 [1] has provided nurses with greater freedom to support GPs and has provided staff with a new framework for organizing and delivering primary care nursing services to improve patient care. Traditionally, GPs have been seen as the primary care gatekeepers, providing a point of access to secondary care, but recent changes have meant that this is now also included within the role of the Primary Care Nurse Practitioner (NP). Until 2002, there was some ambiguity about the defining characteristics of a NP, who as well as being present in primary care settings, have developed roles in secondary and tertiary care settings in a variety of specialities, such as acute care, paediatrics, geriatrics and occupational health. Now, the Royal College of Nursing accreditation units have several standards for NP education in the UK and have defined the domains of clinical competencies [2]. Although this does not include the right to prescribe, in March 2005, the NHS (National Health Service) Modernisation Agency and the Department of Health clarified the regulations on prescribing, thereby allowing nurses and other professionals to prescribe independently of GPs [3]. It has been argued that in England Advanced NPs and NPs are increasingly replacing the role of doctors so that they – rather than doctors – will be the first port of call for most patients [4].

## Methods

A retrospective analysis was conducted using the notes and history of all patients who attended the eye casualty clinic at Princess Margaret Hospital in Swindon during two weeks in March 2006. Usually there was only one doctor in the ECC who may receive assistance from other doctors if the casualty clinic was overwhelmed by patients or if any cases required admission to the ward. The cases which an ophthalmic NP can manage in our department are acute and chronic lid inflammations like stye, chalazion and blepharitis, dry eye, corneal abrasion, corneal and sub tarsal foreign bodies, conjunctivitis, trichiasis and sub conjunctival hemorrhage. Diagnosis was broadly grouped into 5 main groups, namely inflammation/infections, traumatic, vitreo retinal, vascular and miscellaneous: a detailed breakdown of these main groups was made. The Statistical Package for the Social Sciences, version 13 (SPSS Inc, Chicago, IL, USA) was used for statistical analysis.

### We collected the following information from each set of notes

• Date of attendance

• Age

• Gender

• Source of referral

• Principle diagnosis

• Whether patient should have been seen in general clinic

• Whether it was an appropriate casualty review

• Whether the patient was discharged or followed up

• Whether the patient could have been managed by a nurse practitioner

## Results

Our data was obtained from 192 patients who made 209 visits to the Eye Casualty Clinic at Princess Margaret Hospital in Swindon. The average daily attendance was 21 patients who were seen between morning and afternoon sessions in the ECC.

This study included112 (54%) females and 97 (46%) males. The median patient age was 50 years with an age range between 1 and 91 years.

The eye sheets in the patient notes were not available for 10 (4.8%) visits. Of the remaining 199 visits, 80 (40.2%) patients were discharged and 119 (59.8%) were followed up either in the casualty clinic, general clinic, retinal clinic, laser clinic or were admitted.

**Figure 1:**
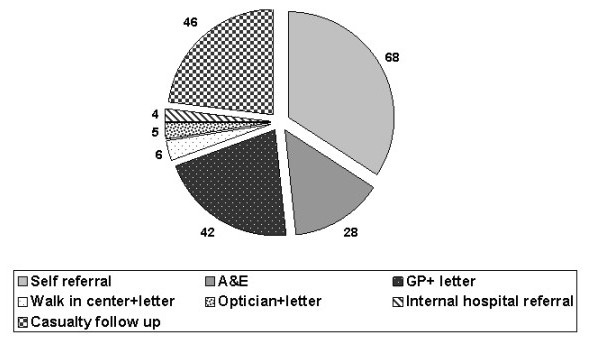
The different sources of referral.

It was found that 68 (34.2%) patients attended as a self referral with no letter as our eye casualty clinic is open to the general public from 9.00 a.m. to 5.00 p.m. A & E referred 28 (14.1%) patients of which only 3 had a General Practitioner (GP) letter and only 1 patient had a walk-in centre letter, figure (1). Appropriate casualty follow-up was made for 46 patients (23.1%). It was found that 42 (21.1%) patients were referred from their GP with a letter, 6 (3.0%) patients were referred directly to eye casualty with a letter from the Walk-in Centre, and 5 (2.5%) patients were referred from their opticians with a letter. Internal hospital referral was made for 4 (2.0%) patients.

**Figure 2:**
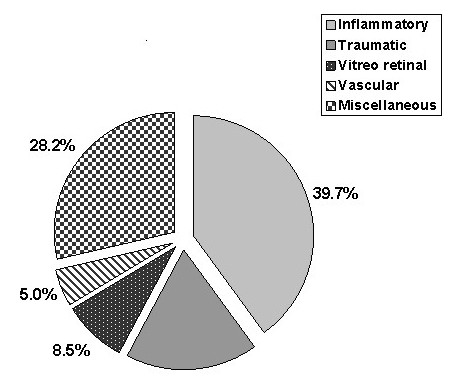
**The main diagnosis of the study subjects**.

Inflammatory/Infection cases were the most common diagnostic group and constituted 79 (39.7%) of patients. figure (1). Trauma was the presenting complaint in 35 (17.6%) patients. Table 1 summarizes all the traumatic cases included in our audit, figure (1). There were 10 (5%) patients presented with vascular problems, the commonest serious problem was central retinal artery occlusion which was seen in 3 patients. (Table 1), figure (1). Miscellaneous conditions were accounted for 56 (28.2%) patients, figure (2). There were 8 cases presented with neurological problems. Table 2 categorizes the miscellaneous problems.

**Table 1:** Classification of cases according to the type of eye disease.

Inflammatory/Infective [n = 79 (39.7%)]	
Conjunctivitis	20
Uveitis	20
Acute eyelid inflammation	1
Chronic eyelid inflammation (blepharitis + chalazion)	7
Episcleritis	6
Scleritis	1
Dendritic ulcer	4
Viral keratitis (Disciform)	3
Dermatitis	1
Marginal keratitis	4
Bacterial corneal ulcer	7
C L related keratitis	2
Preseptal cellulites	3

Traumatic [n = 35 (17.6%)]	

Corneal foreign body	3
Corneal abrasion + erosion	16
Conjunctival foreign body	1
Blunt injury	4
Hyphema	3
Traumatic Iritis	1
Chemical Injury	6
Subconjunctival Hemorrhage	1

Vitreo Retinal [n = 17 (8.5%)]	

Posterior vitreous detachment	11
Retinal detachment	1
Retinal tear	3
Age related macular degeneration	2

Vascular [n = 10 (5%)]	

Central retinal artery occlusion	3
Central retinal vein occlusion	1
Branch retinal vein occlusion	2
Anterior ischemic optic neuropathy (non arteritic)	1
Anterior ischemic optic neuropathy (arteritic) + giant cell arteritis	2
Amaurosis fugax	1

**Table 2:** Categories of the miscellaneous problems.

Miscellaneous n= 56 (28.2%)	12 cases
Post operative problems	
(11 patients following phacoemulsification, of these 4 were routine planned visits and 7 attended with acute problems and walked in and 1 patient following Keratoplasty)	
**Glaucoma**	8 cases
Acute angle closure glaucoma (3 cases)	
Open angle glaucoma (1 case)	
Glaucoma suspect (2 cases)	

**Neurological**	8 cases
Optic neuritis (2 cases)	
Benign intracranial hypertension (1 case)	
Nystagmus (1 case)	
Functional visual loss (2 cases)	
Headache (1 case)	
Migraine (1 case)	

**Corneal abnormalities**	

**Lacrimal**	5 cases
Dry eye (4 cases)	
Epiphora (1 case)	

**Lid**	4 cases
Lid lesion for biopsy (1 case)	
Lid cyst (1 case)	
Entropion (2 cases)	

**Lens**	3 cases
Cataract (1 case)	
Posterior capsule opacity (2 cases)	

**Others**	8 cases
Allergy to glaucoma drops (1 case) (cosopt eye drops)	
Refractive error (1 case)	
No abnormality detected (6 cases)	

There was insufficient information to assess whether 14 patients could have been managed by a nurse; of the remaining 195 visits, 50 (25.6%) patients could have been managed by an Ophthalmic Nurse Practitioner and 145 (74.4%) patients could not have been managed by an Ophthalmic Nurse Practitioner.

The results showed 24 (12%) patients that should have been seen in the General Clinic as they did not have acute problems (follow-up of glaucoma patients, gradual visual loss which leads to cataract, epiphora, follow-up visits for patients with posterior vitreous detachment). Table 3 summarizes these conditions.

**Table 3:** The diagnosis of cases.

Diagnosis	No. of cases (n = 24) (12%)
Routine 2 week follow-up of phacoemulsification	4
Follow-up glaucoma patients	5
Cataract	1
Posterior capsule opacification	2
Chalazion	2
6 week follow-up of posterior vitreous detachment	2
Followup of laser to retinal tear	1
Followup of routine age related macular degeneration	1
2 month follow-up of anterior ischemic optic neuropathy	1
Xanthelasma	1
Refractive error	1
Nystagmus	1
Keratoconus	1
Epiphora	1

## Discussion

The eye casualty clinics are always busy and overburdened. The average number of patients attending our clinic daily is 21; even higher rates are reported in other places [5].

Patients attending ECC with non-acute problems is an ongoing problem that has resulted in an increase in the number of patients attending the casualty clinic. Furthermore a lack of spaces to book reviews into the ECC has also increased. Few ways of addressing this problem include improved ophthalmic training of general practitioners, diverting a greater proportion of non-acute cases to the primary care clinic and expanding the role of the NP [6].

Our basic aim is to provide immediate, maximum attention and care to our attendance of acute cases but in our audit we found that 24(12%) of patients should have been seen in the General Clinic as they were showing signs of non-acute problems. This reduced the slots available for the casualty clinic. Similar results were obtained by other studies such as Jones et al [7] who found that 20% of the eye casualty clinic patients had conditions which could have been dealt with an eye outpatient clinic.

The effectiveness of the eye dedicated nurse practitioner (NP) has been investigated previously and confirmed the high standard of diagnostic and management skills of the nurse practitioner. In a study by Banerjee et al [6] stated that 16.67% of cases seen in eye casualty were managed by a NP, of these the supervising doctor agreed with the diagnosis in all cases and with the proposed management in 96% of cases. Discrepancies were minor and included whether or not to use eye pad (1 case) and the frequency of topical lubricant (1 case).

It was found that 50 (25.1%) patients in our study could have been seen and managed by a competent ophthalmic NP. There are no present reservations by the general public about treatment by a nurse – indeed the patient seems to appreciate the faster service [7], they stated that in total the NP were responsible for 34% of all consultations.

So in a busy casualty clinic like ours, we recommend expanding the role of the NP to serve in the eye casualty clinic at least every morning session to start with. This will give the doctors more time to manage complicated cases and will speed up the service in the casualty clinic without compromising the quality of care.

These are the cases which an ophthalmic NP can manage; acute and chronic lid inflammations like stye, chalazion and blephariti, dry eye, corneal abrasion, corneal and sub tarsal foreign bodies, conjunctivitis, trichiasis and sub conjunctival hemorrhage.

It was found that 68 (34.2%) of our patients were self-referred with no letter from their GP. This number was higher (56%) in Leicester [8] and nearly 80% from Bristol [9]. It can be explained that our casualty clinic is located in the general eye clinic. There were 42(21.1%) patients presented in our ECC with GP letter and 28 patients (14.1%) were referred to us from A & E. The majority of these had no letter. 3 patients had GP letters, 1 patient had a walk-in centre letter and 5 (2.5%) patients were referred with a letter from their Optician.

Fenton et al [10] concluded that 54% of patients were self-referrals, 20% had a previous visit to the hospital, 17% were referred from GP, 5% from another hospital and 3% from Opticians.

We obtained similar results for patients who had previously visited the ECC (23.1%).

In our study (39.6%) patients were discharged whereas Fenton et al in 2001 stated that 56% of their patients were discharged at the first visit. This means that we are asking more patients to come for follow-up appointments in the casualty clinic and this may be due to the type of cases presented to us during the audit period.

At the moment there is no system for patient prioritization in the eye clinic. As a result, it can lead to increased waiting time for some urgent cases which can be lost among a large number of patients waiting in a busy casualty clinic. This made us create ophthalmic triage system in our casualty clinic which will require adequate training for the Ophthalmic nurses to examine the patients and to take the history, accordingly placing the patient in the appropriate category of urgency. Clear guidelines for this will be required for each color coded triage and patients will be categorized into four main groups.

**Red Category**; will be reserved for patients who have to be seen immediately due to penetrating eye injuries, complete loss of vision over the previous 48 hours, alkali burn, children under 3 years with eye problems and total hyphema.

**Yellow Category**; patients should be seen within 30 minutes of their arrival to eye casualty due to ocular pain, requiring admission, partial loss of vision, corneal or conjunctival foreign bodies and blunt injuries to their eyes. In addition patients who are coming for follow-up appointments.

**Green category**; all other patients who come with symptoms of iritis, painful eye conditions like episcleritis, corneal ulcer, diplopia and blurred or distorted vision of recent onset (48 hours) should be seen within 1 hour of their arrival.

**White category**; other conditions which should have been seen in general clinic, sub conjunctival hemorrhage, chalazion and symptoms of conjunctivitis, will be seen last [2].

To enable our casualty clinic to run more swiftly, there are 3 ways to achieve this;

Firstly to decrease the number of patients attending the ECC. This can be done by ensuring that GP and medical staff in A & E know how to deal with the non-acute eye cases and the other acute eye problems which they can manage such as conjunctivitis and foreign body.

Secondly, ensuring that all the follow-up cases of open angle glaucoma and 2 week follow-ups of phacoemulsification will be seen in general clinic.

Lastly, expanding the duties of the nurse practitioner to manage patients with some eye problems and this will decrease the work load in the casualty clinic.

## Conclusion

The workload of the eye casualty doctors could be decreased by 38.6% if defined categories of patients were managed by the ophthalmic nurse practitioner, appropriate referrals were directed to the General Clinic and casualty patients were not followed up inappropriately.

## Recommendations

Expand the role of the Ophthalmic Nurse Practitioner, as it is a cost effective means of reducing the waiting time without compromising the standard of patient care.

Writing of departmental guidelines for some controversial urgent ocular conditions such as optic neuritis and posterior vitreous detachment.

Introduction of Ophthalmic triage system will ensure a proper patient prioritization.

Training of the A & E medical staff and GPs in the use of the slit lamp and management of some eye conditions like red eye, removal of corneal foreign body, corneal abrasion, subconjunctival hemorrhage, acute and chronic lid infections and chalazion, will prevent misutilization of the eye casualty clinic.

## Competing interests

The authors declare that they have no competing interests.

## Authors' contributions

EW participated in the clinical work and the manuscript writing, RS participated in the clinical work in the clinic and data collection, EP participated in the clinical work in the clinic and data collection and AAA-E participated in writing the final manuscript, data collection, data management and gave important clinical suggestions for patient care and management.

All authors read and approved the final manuscript.

## References

[B1] Department of Health Liberating the Talents Helping Primary Care Trusts and Nurses to Deliver the NHS Plan 2002 Dpartment of Health, London

[B2] Royal College of Nursing Practice Nurses and Nurse Practitioners, Recommended Pay, Terms and Conditions 2004 Royal College of Nursing, London

[B3] NHS Modernisation Agency and the Department of Health Medicines Matters, A Guide to Current Mechanisms for the Prescribing, Supply and Administration of Medicines 2005 Department of Health, London

[B4] St Bartholomew School of Nursing & Midwifery_Super Nurses_ are Replacing Doctors and their Advanced Role must be formally Registered, Argues Leading Nurse Academic 2002 Press Office, 25 November, 021/B, City University, London

[B5] Jones NP Hayward JM Khaw PT Claoué CM Elkington AR Function of an ophthalmic "accident and emergency" department: results of a six month survey Br Med J (Clin Res Ed) 1986 18;292 6514 188 190 10.1136/bmj.292.6514.188 PMC1339051 3080128

[B6] Banerjee S Beatty S Tyagi G Kirkby R The role of ophthalmic triage and the nurse practitioner in an eye dedicated department Eye 1998 12 880 882 10070528 10.1038/eye.1998.222

[B7] Jones N Hayward J Khaw P Claoue C Elkington A Function of an ophthalmic "accident and emergency" department; results of a six month survey British Medical Journal 1986 292 188 190 3080128 10.1136/bmj.292.6514.188 PMC1339051

[B8] Chiapella A Rosenthal R One year in eye casualty clinic British Journal of Ophthalmology 1985 69 865 870 4063253 10.1136/bjo.69.11.865 PMC1040761

[B9] Vernon SA Analysis of all new cases seen in a busy regional centre ophthalmic casualty department during 24 week period Journal of the Royal Society of Medicine 1983 76 279 282 6601713 10.1177/014107688307600408 PMC1438967

[B10] Fenton S Jackson E Fenton M An audit of the ophthalmic division of the accident and emergency department of the Royal Victoria Eye and Ear Hospital, Dublin Ir-Med-J 2001 94 9 265 6 11820516

